# Fostering social innovation and building adaptive capacity for dengue control in Cambodia: a case study

**DOI:** 10.1186/s40249-020-00734-y

**Published:** 2020-09-03

**Authors:** Pierre Echaubard, Chea Thy, Soun Sokha, Set Srun, Claudia Nieto-Sanchez, Koen Peters Grietens, Noel R. Juban, Jana Mier-Alpano, Sucelle Deacosta, Mojgan Sami, Leo Braack, Bernadette Ramirez, Jeffrey Hii

**Affiliations:** 1 SOAS University London, Thornhaugh Street, London, WC1H 0XG UK; 2 Malaria Consortium, Phnom Penh, Cambodia; 3 Institute of Tropical Medicine, Antwerp, Belgium; 4 Social Innovation and Health Initiatives, University of the Philipines, Manilla, Philippines; 5 California State University, Fullerton, USA; 6 UNICEF/UNDP/World Bank/WHO Special Programme for Research and Training in Tropical Diseases, Geneva, Switzerland; 7 Australian Institute of Tropical Health & Medicine, James Cook University of North Queesland, Townsville, QLD Australia

**Keywords:** Social-ecological system, Community engagement, Transdisciplinarity, Health development, Sustainability, Integrated vector management, Social innovation in health

## Abstract

**Background:**

The social-ecological systems theory, with its unique conception of resilience (social-ecological systems & resilience, SESR), provides an operational framework that currently best meets the need for integration and adaptive governance as encouraged by the Sustainable Development Goals. SESR accounts for the complex dynamics of social-ecological systems and operationalizes transdisciplinarity by focusing on community engagement, value co-creation, decentralized leadership and social innovation. Targeting Social Innovation (SI) in the context of implementation research for vector-borne diseases (VBD) control offers a low-cost strategy to contribute to lasting and contextualized community engagement in disease control and health development in low and middle income countries of the global south. In this article we describe the processes of community engagement and transdisciplinary collaboration underpinning community-based dengue management in rural primary schools and households in two districts in Cambodia.

**Methods:**

Multiple student-led and community-based interventions have been implemented focusing on empowering education, communication for behavioral change and participatory epidemiology mapping in order to engage Cambodian communities in dengue control. We describe in particular the significance of the participatory processes that have contributed to the design of SI products that emerged following iterative consultations with community stakeholders to address the dengue problem.

**Results:**

The SI products that emerged following our interaction with community members are 1) adult mosquito traps made locally from solid waste collections, 2) revised dengue curriculum with hands-on activities for transformative learning, 3) guppy distribution systems led by community members, 4) co-design of dengue prevention communication material by students and community members, 5) community mapping.

**Conclusions:**

The initiative described in this article put in motion processes of community engagement towards creating ownership of dengue control interventions tools by community stakeholders, including school children. While the project is ongoing, the project’s interventions so far implemented have contributed to the emergence of culturally relevant SI products and provided initial clues regarding 1) the conditions allowing SI to emerge, 2) specific mechanisms by which it happens and 3) how external parties can facilitate SI emergence. Overall there seems to be a strong argument to be made in supporting SI as a desirable outcome of project implementation towards building adaptive capacity and resilience and to use the protocol supporting this project implementation as an operational guiding document for other VBD adaptive management in the region.

## Background

The United Nations (UN) 2030 agenda for sustainable development and its Sustainable Development Goals (SDGs), aim to provide a comprehensive blueprint for human development by recognizing that opportunities to improve health can be found not only in specific health interventions (principally in SDG 3), but also through social justice (SDGs 4, 5, 10, 16–17), environmental protection (SDGs 2, 6, 7, 11–15), and shared prosperity (SDGs 1, 8, 9). There is a strong crossover between the SDGs and the social determinants of health, as elaborated in the Alma Ata declaration and later strengthened by the launch of the Commision on Social Determinant of Heath [1]. There are also significant cross-overs with the Social ecological systems theory and its unique conception of resilience applied to health emphasizing the interdependence of human society and nature and supporting adaptive governance in this regard [2, 3].

The UN 2030 agenda and the SDGs thus provide a further, perhaps more compelling incentive and opportunity to operationalize integration across traditional disciplinary and sectorial silos and domains of development. However, overcoming the challenge of integration and cross-sectorial collaboration in development, central to the SDGs, is particularly difficult as the social-ecological systems within which integrated development actions are elaborated are subject to constant change, requiring development modalities and collaborations to be adaptive and frequently revised. Accordingly there is a need for a contextualized balance between government-led policy decisions and community-based decentralized leadership in order to ensure timely and culturally adapted (and adaptive) interventions.

The social-ecological systems theory, with its unique conception of resilience (social-ecological systems & resiliance, SESR), provides a framework that currently best meets this need for adaptive governance and accounts for the complex dynamics of social-ecological systems [3]. Originally developed on the basis of studies of ecosystem dynamics, SESR has grown into a robust integrative, transdisciplinary approach that uniquely combines natural and social sciences perspectives. As a central postulate and heuristic tool SESR’s adaptive cycle has proven widely applicable for understanding adaptation and sustainability across different types of systems [3]. As it is based on principles emerging from studies of ecosystem functioning applied to sustainable resources management and development, it is particularly applicable to complex problems at the human-animal-environment interface, especially emerging zoonoses [4]. SESR and its heuristic metaphor, the adaptive cycle, emphasize the importance of building adaptive capacity to support system’s resilience - the capacity of a social-ecological system to absorb or withstand perturbations and other stressors allowing it to maintain its structure and functions (i.e., does not undergo collapse and regime change). This requires a transdisciplinarity process [3, 5] including community engagement, value co-creation, decentralized leadership and social innovation.

Social innovation (SI) is a process of developing and deploying effective solutions to challenging and often systematic environmental issues in support of social progress. SI focuses attention on the ideas and solutions that create social value—as well as the processes through which they are generated, regardless of where they are coming from.^1^

Although in its infancy as a science, social innovation in health (SIH^2^) can be seen as an important part of communities’ adaptive capacity through encouraging communities and individuals to be active interpreters of their lives and essential contributors in solving creatively the health challenges that they face (i.e. not just passive beneficiaries [6]). As such, SIs could be targeted as desirable attributes of public health project’s outcomes sustainability and communities’ resilience. Following this rationale, the Social Innovation in Health Initiative launched by TDR in 2014^3^ is intended to provide leadership to advance social innovation for communities challenged with infectious diseases with the ultimate goal of achieving the SDGs. SI applied to the control of VBDs offers an opportunity for more precise problem framing as a basis for intervention research including a focus on grass-roots innovation in low and middle income countries of the global south [7].

In this article we describe an ongoing effort to engage Cambodian communities and schools in dengue control and describe experiences and lessons-learnt through project implementation. The intention of this article is not to extensively present qualitative results of the empirical research conducted so far but to offer a perspective on what a more integrated community-based VBD adaptive management effort could look like. We particularly focus on the significance of the participatory processes that have contributed to the design of SI products that emerged following iterative consultations with community stakeholders to address the dengue problem. We also discuss the parallels between SIH and “empowering health education” as well as the significance of social innovations and social entrepreneurship for continued community engagement, adaptive capacity building, and sustainable health development.

## Methods

### Dengue and dengue control in Cambodia

Dengue is the most rapidly spreading mosquito-borne viral disease in the world and is strongly related to urban expansion worldwide, particularly in tropical regions [8]. Dengue is caused by bites of infected *Aedes* mosquitoes, principally *Aedes aegypti* [9]. Asia records 70% of the global disease burden due to dengue [10], and Cambodia has one of the highest per-capita incidence rates in the region [11]. Identified in Cambodia in 1963 [12] a total of 194 726 dengue cases were reported to the National Dengue Control Program (NDCP) between 1980 and 2008 [13]. Between 2003 and 2008, annual dengue incidence ranged between 0.7 and 3.0 per 1000 persons, the cost to society estimated at between USD 3 327 284 and USD 14 429 513 [14]. Since most of this cost falls onto the family, it is estimated that 67% of affected households fall into debt to pay for medical bills [15]. However, as many components of dengue transmission remains unclear, the real number of cases and cost to society is likely much greater, with some studies suggesting the real case numbers are between 3.9 and 29.0 times higher than those of the National Dengue Surveillance System [16, 17].

Dengue vector control in Cambodia relies on disease surveillance using existing health reporting systems, emergency preparedness and outbreak containment, health education, mass temephos larviciding in high risk provinces and clinical management. The impact is variable, and endemic dengue transmission persists annually due to management, resource and operational issues as well as increasing temephos resistance [18]. Health education for dengue control is provided in primary schools, at village health centers (HCs), and by the NDCP. However, these educational programs are accorded low priority, strategies do not consider existing evidence, materials are not evaluated on a routine basis, messages are not validated with local communities, and health staff and teachers lack training, communication skills, time and opportunities to deliver educational messages [19]. Recommendations to villagers are not always practical or effective in preventing mosquito bites, and funds are not available to provide new educational materials [19]. While school children and their parents have some familiarity with the behaviour and habitat of the *Aedes* mosquito and the environmental factors that contribute to dengue fever, their knowledge is uneven and knowledge is rarely translated to reduce the risk of infection. It is therefore critical to engage with these communities and ensure health education is regularly resourced in innovative ways, and to ensure that lessons on prevention result in concrete actions relevant to resources level and cultural acceptability.

Novel approaches to dengue control have been implemented recently in Cambodia, including a large-scale community-based larvivorous guppy fish (*Poecilia reticuluta*) distribution complemented with Communication for Behavioural Impact (COMBI) [20]. The outcomes of these projects were encouraging as guppy coverage increased and acceptance by community members was high (88%) and resulted in significant decline in larval-infested breeding containers (container index) (92.5%) [20]. This was followed by a randomised controlled trial (RCT) of guppy and pyriproxyfen distribution (supported by a bottom-up COMBI planning process) in Cambodia which gave 53 and 44% reduction, respectively, of *Aedes* adult mosquitos as compared to the control group [21, 22]. A well-informed COMBI strategy and high community participation and ownership resulted in high acceptance of guppy fish in the intervention villages, and a high preference for guppy fish over other insecticide-based methods due to their ease of use and rearing, quick reproduction, propensity to eat larvae and sustainability [23]. Furthermore, researchers and local program managers believe that the combination of guppy and autocidal gravid ovitraps (AGO) can potentially result in further significant vector reduction through the reduction of the abundance of potentially infected (gravid and parous) females of *Ae. aegypti*, leading to a significant and sustained reduction in disease transmission particularly if implemented in both schools and communities [24–26]. Based on the lessons learnt from these two RCT guppy distribution projects [27], there is a clear opportunity to roll out and integrate these low-cost, year-round tools into the school-health curriculum and local community groups so they can manage local guppy breeding and distribution programs. By doing so, decentralized surveillance capacity and robust, community-led dengue control operations in an area wide vector control program are more likely to happen and be sustained.

### Project design and implementation strategy

Following the conceptual and methodological rationale offered by SESR [3]—particularly the importance of community engagement and adaptive capacity building towards learning and social innovation— and recognizing the existing readiness of communities in Cambodian endemic areas to participate in community-led control activities [28], the project aimed at operationalizing an integrated vector management (IVM) program through community-based distribution and monitoring system of AGO traps in conjunction with ongoing school based rearing and distribution of guppy fish to communities. Accordingly, the randomized control trial investigated whether a set of interventions, including IVM-based source reduction procedures [29], COMBI-based health experiential education and community engagement, could significantly reduce dengue entomological indicators in rural primary schools and households and contribute to community adaptive capacity in two districts in Kampong Cham, Cambodia. (Table 1).

**Table 1:** Interventions randomized to each study arm

Intervention type	Intervention component	Intervention short description	Arm 1		Arm 2		Arm 3	
			Schools	Village	Schools	Village	Schools	Village
**Biophysical**	**Vector control**	Autocidal gravid ovitraps (AGO)	✓	✓	✓	✓		
	**Biological control**	Guppy distribution	✓	✓	✓	✓		
	**Solid waste management**	Larval source control through improved solid waste management	✓	✓	✓	✓		
**Empowerment/Adaptive-capacity**	**Education and training**	Place-based educational campaign on dengue disease; vector biology; ecology, and control; role of solid waste, clean water & health relationships	✓	✓				
	**Communication & Behaviour change**	Communication for Behavioural Impact using multipronged communication channels including interpersonal communication through volunteers, folk or local media and mass media.	✓	✓				
	**Participatory mapping**	Map co-creation as a tool for community ownership of dengue decentralized surveillance and management	✓	✓				

To achieve community ownership and empowerment (sensu [30]) regarding to the use of vector control tools, the project implementation involved community-participatory methodologies and capacity-building activities at critical stages of the project implementation. These included engagement of teachers, school directors and ministry of education representatives in the redefinition of the curriculum for dengue health education, students’ involvement in the construction of key strategies and messages to be distributed at community and school levels, and community definition of the implementation channels of the proposed solution. As the proposed project planned to scale up existing community-based dengue vector control approaches, for which there is already extensive and contextualized experience, the project team has looked into the needs, expectations, concerns, desires and knowledge in relation to health of the Cambodian communities and stakeholders from various sectors. Motivated, empowered and well-informed multi-stakeholder and gender-diverse groups, whose area of influence span multiple administrative and institutional scales, should be better able to identify and sustainably implement adaptive dengue control strategies, as they are better equipped to understand the tools available to them and mitigate cross-scale social and ecological drivers of disease emergence [31, 32]. Through its participatory activities and empowering interventions, the project deliberately focused on building adaptive capacity through key community and school driven social innovations.

## Results

In the first phase of implementation as well as post intervention, formative research [33, 34] was conducted and qualitative assessment were performed. In-depth interviews, focus group discussions, participant observations, and informal conversations were conducted with several levels of stakeholders and actors at the community level, including community health workers (CHWs), health centre chiefs, school directors, monks, priests, teachers, farmers and members of the local education office as well as students.

Qualitative data collected has been used to facilitate community dialogues and inform workshops focused on messages and material’s design within the aforementioned COMBI strategy. These workshops, in the form of group discussions, were held with key community members, volunteers and district stakeholders in schools included in intervention arms. During these meetings, participants agreed that vector control tools needed to be adapted to the community context and integrated into the schools’ health education revised curriculum. In fact, there was a general sense of agreement towards strengthening the project’s “adaptive capacity building/empowerment/educational” aspects and the qualitative dimensions of the project. An emerging question that arose during these community gatherings is how can we use vector control tools that most community members agree are efficient (i.e. guppies) to revamp the health education curriculum and integrate these tools for routine communication in community settings. In summary, there was a general demonstration of interest by community actors to improve ownership of the vector tools and a strong participation towards adapting tools and methods to the cultural context and local socio-economic level. This indicates that SI, a processes through which social change grounded in local realities is generated, has emerged from the engagement of stakeholders during the numerous meettings, workshops, focus group discussions and interviews. Together with SI, several social innovation products were developed, representing critical project outputs towards building community adaptive capacity and project outcome sustainability. These SI products are described below.

- *Locally made adult mosquito traps from solid waste collections.*

**Fig. 1:**
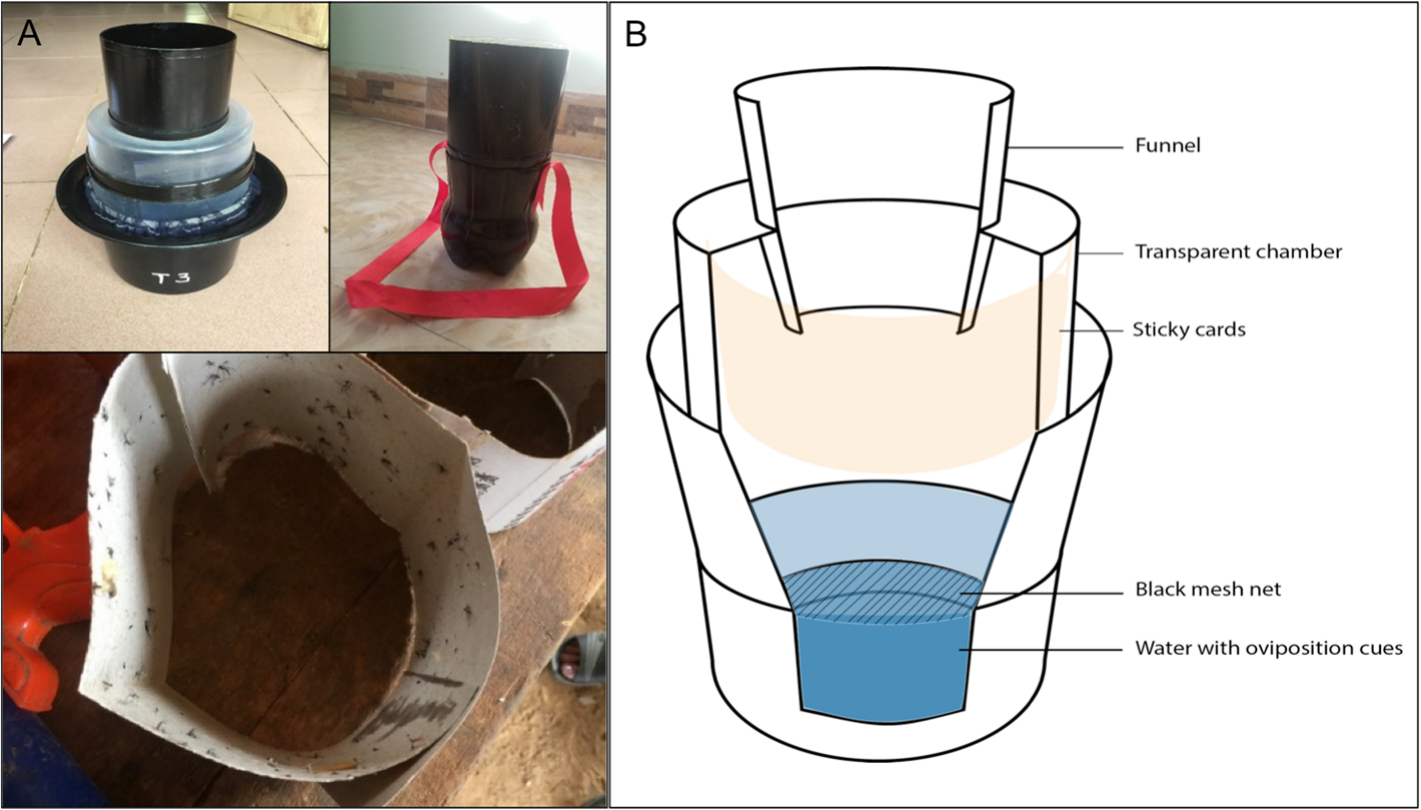
Locally made adult mosquito traps replicating autocidal gravid ovitraps (AGO). **A** Finished product, **B** Schematic design. The AGO trap consists of five basic components: 1) black polyethylene cylinder that serves as the trap entrance (12.8 cm in diameter) and transparent capture chamber; 2) sticky surface covering the interior of the capture chamber that is coated with 155 g/m^2^ of a nonsetting polybutylene adhesive 3) screen barrier at the bottom of the capture chamber to prevent adult mosquitoes from moving between the capture chamber and the infusion reservoir. It also prevents any mosquito emerging from the infusion to escape from the trap (occasionally, eggs from captured females may be washed by rain into the infusion reservoir and develop into adult mosquitoes); 4) black polyethylene pail with drainage holes to allow excess infusion to drain from the trap 5) infused water

Through regular visits and collaborative trainings, women’s groups were capable of producing 3228 medium size traps (MST) and 6300 small size traps (SST), a total production of 9528 traps. These traps which replicate autocidal gravid ovitraps [35] in principle (Fig. 1), were placed in 20 implementation villages with 3 traps (1 MST + 2 SST) deployed per household (HH) in each of the 3158 households, and 2 traps (2 MST) in each of the 161 rooms in 16 schools. Beyond the impact on entomological indicators, the process of developing the trap design with the women’s group during workshops and subsequent follow-ups contributed to a community-owned innovation and an increased sense of ownership of the product and its use (Additional file 1). This was demonstrated through the ongoing transformation of the women’s group from casual gatherings to civil society organizations and social enterprises. These more formal organizations are being created with the intention to improve the design and distribution of the traps as well as to develop outreach strategies for continuing impact. In addition to that, the traps, made of recycled plastic bottles, generated income for the participants and contributed to increase awareness regarding solid waste management and effectively recycling plastic waste. Together these actions and outcomes encourage positive change and offer new opportunities.

- *Dengue curriculum with hands-on activities for transformative learning*

The team facilitators worked closely with the Ministry of Education and the School Health Department of the Ministry of Health as well as school directors, officers and teachers to collaborate on the revision of the health education curriculum to incorporate elements of Dengue transmission, mosquito biology and ecology, biocontrol with guppies, waste management to minimize breeding sites as well as mosquito collection procedures (Table 2). The project team facilitated training sessions that also included pedagogy, learning and teaching style focusing on hands-on transformative learning experiences. These training sessions provided the basic material, know-hows and inspiration for teachers to subsequently implement the revised curriculum and hands-on activities with maximum engagement of the students. In total about 100 teachers, school directors and officers participated in these training sessions and over 500 students were involved in receiving and in turn communicating this novel curriculum content. For students, part of the transformative learning experience was related to their contribution to community-based “education” sessions whereby students could showcase their acquired understanding of dengue, its significance and how to address the problem in their communities. Students participated in 40 of these sessions during which knowledge transfer across generations was meant to augment current community sense of ownership of dengue and its control (Additional file 2).

**Table 2:** Porposed learning activities to be part of the co-designed curriculum

Project vector control interventions	Specific vector control activities	Educational activities	Learning objective	Hands-on practice
A. Vector Control	A1. Traps	1. Mosquito traps construction	1. Learning how autocidal gravid trap work	1. Visual representation of the autocidal gravid ovitraps (AGO) and its functioning, illustration of mosquito behavior 2. Procedure to make locally and cheaply the AGO
		2. Mosquito trap distribution	1. Learning how to make, distribute and monitor autocidal traps in schools and households	1. Design and illustrate the “AGO value chain” from the supply of the trap to its distribution 2. hands-on practice for distributing traps in schools and households
	A2. Solid waste management and environmental assessment	3. Life skills: the 3Rs	1. Life skills: Learning the basic 3Rs: reduce, reuse, recycle. Learning how we can reduce the use of single use plastic containers	1. Visual description of 3Rs 2. Applications/example to real life of students
		4. Life skills: waste management	1. Life skills: co-design simple sustainable waste management strategies in schools and households to reduce plastic containers and mosquito breeding habitats	1. School and household premise exploration and problems identification (when and why are waste produced? 2. Visit of landfill/recycling factory 3. School and household plan for waste reduction and safe disposal
		5. Container cover	1. Learn what containers are good breeding site for mosquitoes 2. Learn why these containers are used and what alternatives could be proposed	1. School and household surveys to list containers types and functions 2. Develop mitigation measures including cover types and cover check up surveys
		6. Vector Habitat modification	1. Learn how to identify various mosquito breeding habitats and how to reduce their occurrence	1. Visit of school premises and identification of breeding sites Define solutions with student to reduce breeding site (e.g. fill up ditches)
	A3. Biocontrol	7. Guppy life cycle	1. Learning what are guppies, how they live and what they eat? 2. Learning how to take care and become responsible	1. Rearing experiment and observationDrawing of guppy life cycle
		8. Other biocontrol agents	1. Learning what other organism could also help reducing mosquito abundance 2. Learning how to design simple “research”(what to ask, who to ask and where to find the information	1. Interviews with fishermen and elders in the community 2. Web search
		9. Food chain	1. Learning the interrelations of living organisms 2. Learning basic experimental design	1. Food chain observations during nature walk (simple observation: herbivores, carnivores) 2. Guppy-mosquito predator prey experiments
		10. Guppy bank	1. Learning how to rear and take care of guppies 2. Become responsible and learning how to manage guppy banks 3. Learning how to engage with community members	1. Set up of guppy bank 2. Set up a monitoring/caring system (e.g. every week different student take care of the guppies) 3. Community engagement through school-based guppy distribution
B. Surveillance	B1. Vector Data collection and mapping	11. Mosquito collection	1. Learning how to collect adult mosquitoes from traps and larvae and pupae from containers 2. Learning how to identify mosquitoes 3. Learning basic data collection methods	1. Trap monitoring for adult mosquito counting and identification 2. Container screening for larvae and pupae collection and identification as well as mosquito life cycle experiment
		12. Mosquito breeding habitat mapping	1. Learning how to use GPS 2. Learning how to create a map by hand and by using google map	1. School and household transect walk and identification of breeding sites as well as use of GPS 2. Google map sessions
	B2. Mosquito ecology and Identification	13. Mosquito ecology and rapid ID diagnostic	1. Learning basic mosquito features 2. Learning mosquitoes life cycle 3. Learning about mosquito ecology 4. Learning how to identify main vector sp.	1. Design of biology sessions with observations/dissection and drawing 2. Mosquitoes, rearing experiments, identification
C. Communication	C1. Basic communication skills	14. Communication best practices	1. Learning basic communication principles 2. Learning how to use online resources and softwares to help with communication	1. Role playing games 2. Interactive online sessions
	C2. Dengue awareness campaign	15. Design dengue awareness material	1. Learning how to summarize Dengue knowledge 2. Learning how to create communication material	1. Creation of videos, posters and games 2. Creating a Youtube channel a in remote rural areas. 3. Organization of science fair activities and community campaigns

The cross-sectorial collaboration and transdisciplinary action that took place during the school-based sessions together with the strong engagement of students in activities of knowledge sharing in communities, led the department of school health of the ministry of education to incorporate the co-designed dengue curriculum into the national school program with 1 h per week allocated to dengue and its community-based integrated control.

- *The strengthening of guppy distribution systems by community members.*

An essential and very effective vector control tool in this project is the use of guppy fish in water storage tanks as well as smaller containers that are commonly found around households. The efficiency and acceptability of guppies in reducing vector abundance has been demonstrated in several projects including previous community-based dengue trials [28]. The project team together with school partners, community leaders and community health workers established guppy fish banks in schools (3 jars × 16 schools), in community settings (6 jars × 20 communities) and at health centers (20 jars × 6 HCs). Students were involved in guppy fish distribution to their households and community health workers were responsible for distributing guppies to community guppy banks.

Community members could also directly collect guppies from the health centers. A total of 22 400 guppy fishes were distributed in the first 6 months of the project. Training sessions have been facilitated to empower 100 school teachers, 94 CHWs and staff from six health centers providing knowledge on how to rear, maintain and distribute guppy fishes (Additional file 3). Overall there has been a strong consensus of the relevance and ease of use of guppies as “decentralized” vector control tool as described in the following accounts:

*“The guppy fish is not complicated; it is no need to take care of it for often. It can eat all the larvae from the water containers.” Male CHW in Kraloang village, 49 years old.*

*“We distributed three fishes, two females and one male to students, right now there is much fish still in the jars. We still give to students who lost their fish when they ask from us.” Teacher, 20 years old.*

The iterative community engagement initiatives regarding guppy use and distribution has led to a dramatic increase of guppy presence in households, from 11% of HH using guppies in August 2018 to 42% in August 2019 with about 1260 households now having guppies. Observations or anecdotal reports that were received from community members indicated that guppies were informally distributed outside interventions areas, suggesting knowledge transfer, cultural acceptability, strong feasibility of scaling up and project outcome sustainbility. Discussion during intervention follow-up sessions as well as during the November 2019 research uptake meeting highlighted the value of further operationalizing the guppy distribution system. Among the ideas exchanged, it was mentioned that the development of a phone application would offer a flexible interface for communication among distributors and household members or guppy banks in the communities regarding stocks and refill needs and create another opportunity for social innovation.

- *Co-design of dengue prevention communication material by students and community members.*

Focus group discussions and key informant interviews in school and community settings enabled constructive discussions and significant engagement of stakeholders. Participants generally considered that working with schools was a good strategy to introduce knowledge on guppy rearing and care, as well as to bring that knowledge to villages’ homes through involved pupils. However, participants pointed out that working at the village level is equally important:

*“I think we have to do both. The school is the place to grow the human resource for present and the future because they are young ( … ) it is good for them to receive the knowledge. But for the adult people who live in the community, they don't get any knowledge from the children because some children can learn but they cannot explain to their parents ( … ) so we have to do both.” Monk, 38 years old.*

Recommendations about relevant sites for the diffusion of information at community levels included pagodas, commune halls, health centers and private medical practices. The mobilization of health workers during vaccination campaigns was also seen as reference—and potential strategy—for the diffusion of health-related information. Similarly, monks have proposed ceremonial occasions at the pagoda as acceptable moments to communicate dengue related control knowledge or procedure, provided that they previously receive education on dengue control.

In relation to communication channels, women and grandparents were identified as responsible for decision-making and implementation of prevention activities in relation to dengue control at the household level in the past. Participants agreed that women and grandparents should be mobilized as key actors in current and future dengue interventions, particularly regarding enabling knowledge and action to flow from schools to communities through their privileged relationships with their children. Village health workers are also generally trusted as sources of information at the community level, particularly in contexts where health centres’ collaboration with local schools is highly dependant on staff’s availability.

Content wise, most participants were aware that dengue fever is caused by *Aedes*, locally known as ‘tiger’ mosquitoes. Interviewees generally stated that guppy fish, ABATE (Temephos) larvicide and environmental cleaning around their settlement can be useful methods to eliminate mosquitoes breeding sites. Playing spaces around mango trees were referred as potentially high-risk sites for transmission.

Initial communication material was developed during “high level” stakeholder meetings whereby official representatives of government bodies as well as community leaders met to prepare the planning of intervention activities, monitoring and evaluation, and to help to mobilize local resources and give logistical support. It was further adapted during 40 health education sessions where students presented their own versions of the posters and banners. The students communicated their material to an audience of between 20 and 45 villagers in each of these sessions with the support of CHW for the design of specific messages.

- *Community mapping*

An important medium of engagement was the co-creation of community maps spatially representing local perception of breeding sites locations, zones of contact with mosquitoes, frequency, extent and timing of people movement, significant infrastructure enabling mosquito presence and general epidemiological data. About 650 villagers, particularly women, participated in Particpatory Epidemiology Mapping (PEM) sessions and were actively involved in the identification of the dengue transmission arena boundaries (Additional file 4).

During PEM sessions, higher participation from local people contributed to increase local mobilization in reducing breeding sites, leading to reducing the adult mosquito population (manuscript in preparation). The maps created could then be used to focus subsequent vector-control effort and better understand dengue transmission overall. Participants involved in PEM have significantly developed new relationships between their experiences and the knowledge shared during the sessions. Participants could then compare the map to the real infrastructure elements or processes in their village.

*“PEM could help local people to identify and manage the mosquito breeding site in the village. People will be aware of mosquito breeding place around the house and in the village.” Krasaing Pul village chief, 60 years old.*

Participants indicated that PEM was a useful tool for them to know how to identify breeding sites and locate them as well as to help CHWs improve control initiatives in the village. The outputs, mat mapping or paper maps (Additional file 4), can also support primary schools teaching capacity helping students understand the local transmission locations.

The process of mapping and the discussions around it also contributed to highlight knowledge gaps. Most of the participants for instance were still confused and surprised that mosquito larvae aquatic habitats could be found in and around lakes, ponds, or streams. Some people also were not aware of the breeding sites around their house. The majority of participants were female and elders while young men were at work and during participatory session only few outspoken individuals mostly contributed

*“Even there are many participants in PEM but only a few people expressed their idea in the meeting” Male, 35 years old.*

However, as information was discussed the message was heard by all and consensual spatial representations were made.

It was observed that, through the series of community sessions, participants gradually acquired a stronger sense of ownership and the capacity to become the stewards for their own vector control responsibilities as the maps took shape session after session (manuscript in preparation). As such, the process of spatially representing epidemiological information and infrastructure, create a forum for community members to strengthen the community relevance and practicality of dengue and its control. Control and surveillance intervention then become grounded in community context and therefore ownable and actionable. Doing so means that community-based trapping scheme (for surveillance) or school science approach for dengue mosquito monitoring can support government-led dengue vector surveillance and control by providing insight in vector species distribution and dengue transmission local patterns. From our observations, we anticipate that the data generated via these approaches are relevant for the planning, implementation and evaluation of vector control activities by NDCP. The involvement of local schools or communities in the science of mosquito ecology is expected to lead to more sustainable solutions for dengue control. This approach presents opportunities to bring down institutional barriers, such as low level of community involvement in vector control, limited financial resources for mosquito surveillance and the current exclusion of more remote areas in mosquito monitoring which are known to remain critical impediments to sustainable vector control.

## Discussion

Social innovation in health presents a lens or an approach through which countries can be supported to achieve sustainable, equitable and integrated people-centred health systems and health services. Contrary to commercially-focused innovation, the primary intended outcome of social innovation is enhanced quality of life, justice and equity for all members of society [36, 37].

Thus, the social innovation approach could hold the potential to breathe fresh life into the 1978 Alma Ata ideals of equity, social justice and community participation in basic health care delivery [38] and support the achievement of Universal Health Coverage and the Sustainable Development Goals.

Complementarily, SIH approaches aligns with empowerment education, with its emphasis on the collective knowledge that emerges from group sharing experiences, understanding of the social influences that affect individual lifes and identify critically and collectively positive changes that can be made [30]. In the context of this project, where so-called health education was a significant focus, SIH links with a more empowering health education effort that embodies a broad process encompassing prevention as well as other goals of community connectedness, self-development, improved quality of life as well as social and environmental justice [34]. The project implementation phases, including its extensive formative assessment and participatory components, aligns with the conceptual and methodological characteristics of Paolo Freire’s three-steps empowering education program as seminally described in [39] and subsequently adapted in [30] (Table 3).

**Table 3:** Freire’s seminal three-stage methodology for empowering education

Step	Description
1	Listening to understand felt issues or themes of community
2	Participatory dialogue about the investigated issues using a problem-posing methodology
3	Action or the positive changes that people envision during their dialogue

While the project is ongoing and a more definitive assessment of its outputs and outcomes will follow (including post project assessments), we observed that new services, products, financial models, behaviours and policies that are more inclusive, effective and sustainable are already being negotiated among community actors. The systems transforming dimension of social innovation, as supported by project’s interventions, sets it apart from more common forms of innovation. By challenging social practices, rules and social relationships, social innovation and its products specific to this project do more than just address the dengue problem. They provide communities with a heuristic for coping and adapting to new challenges at large, hence broadening the scope of public health to also integrate larger societal and environmental issues and by doing so making communities more resilient.

In the context of this project, the social innovations products that are emerging can be regarded as creative solutions collectively negotiated from the bottom-up with strong women representation and child inclusiveness [40]. Particularly in the case of VBDs this implied understanding local natural and social ecologies as well as identifying culturally adapted tools to act on them in a participatory manner. Accordingly enabling or encouraging SI can be seen as a practical key step towards operationalizing transdisciplinarity as well as a desirable and measurable outcome of SESR approaches towards adaptive governance [3]. SI appears to insure the necessary contextualization of infectious disease management within a more equitable and sustainable health development narrative and improve vector control success during and beyond the life-span of a particular project. For this WHO-TDR dengue vector reduction project in Cambodia for instance, one direct measure of short-term success in community uptake and application of project interventions was the extent to which such interventions resulted in vector mosquito populations reduction. With only two project staff in the project area to coordinate community interventions involving several thousand villagers in multiple villages, the level of community support within 1 year of project inception resulted in a dramatic reduction in all entomological indicators in the intervention villages compared with control villages (manuscript in preparation). As described, the community driven interventions involved acceptance and use of guppy fish for mosquito larval reduction in water storage containers, use of home-made traps for adult mosquito captures, and measures aimed to reduction of mosquito breeding sites around households. Strong reductions in multiple indicators relating to mosquito breeding and abundance, incuding larval, pupal and adult numbers unequivocally point to the advantages and benefits of gaining community understanding and support for public health objectives, and joint planning and implementation of interventions. Not only does this translate into more effective interventions, but also long-term sustainability of such actions.

## Conclusions

As currently provided in Cambodia, dengue health education delivered through health outreach activities and school-based programs is insufficient, under-funded and often irrelevant to the community context [19]. There is a need to engage community stakeholders in the co-design of dengue control interventions that are meaningful to them and, in parallel, design dengue related health education curriculum that are better contributing to transformative learning processes and empowerment.

In Cambodia, there is a need for simple, low-cost solutions to improve health care and to heal the fragmentation between policy-makers and ground-level solutions. This fragmentation has been a long-standing barrier to the implementation of new solutions and suggests there is the potential for social entrepreneurial strategies to bridge the gap between action and policy. The limited time, money, programmes and personnel available to cope with health concerns further point towards the opportunity to bridge the “know–do gap” with the innovative solutions that social entrepreneurs or civil society organizations could pioneer.

The initiative described in this article put in motion processes of community engagement towards creating ownership of dengue control interventions tools by community stakeholders, including school children. While the project is ongoing, the project’s interventions so far implemented have contributed to the emergence of culturally relevant SI products and provided initial clues regarding 1) the conditions allowing SI to emerge, 2) specific mechanisms by which it happens and 3) how external parties can facilitate SI emergence. Overall there seems to be a strong argument to be made in supporting SI as a desirable outcome of project implementation towards building adaptive capacity and resilience and to use the protocol supporting this project implementation as an operational guiding document for other vector-borne disease adaptive management in the region.

## Supplementary information

**Additional file 1.** Trap production system and process of community inclusion. The upper left corner image shows an interaction with a local craftsman helping with traps design adaptation; continuing right and down to the lower rightmost corner, the images show women group’s involvement in production of large enough numbers of traps to be used in intervention schools and communities as well as school children involvement in trap deployment and use.

**Additional file 2.** School-based and student led communication of Dengue information.

**Additional file 3.** Guppy fish distribution network.

**Additional file 4.** Participatory Epidemiology Mapping.

## Data Availability

The datasets generated and/or analysed during the current study are not publicly available in order to respect participants privacy but are available from the corresponding author on reasonable request.

## Abbreviations

3Rs: Reduce, reuse, recycle
AGO: Autocidal gravid ovitraps
CHWs: Community health workers
COMBI: Communication for Behavioural Impact
HCs: Health centers
HH: Household
IVM: Integrated vector management
MST: Medium size trap
NDCP: National Dengue Control Program
PEM: Participatory Epidemiological Mapping
RCT: Randomised controlled trial
SDGs: Sustainable Development Goals
SESR: Socio-ecological Systems & Resiliance
SI: Social Innovation
SIH: Social Innovation in Health
SST: Small size trap
UN: United Nations
VBD: Vector-Borne Diseases
VBDC: Vector-Borne Disease Control

## Footnotes

1 https://www.gsb.stanford.edu/faculty-research/centers-initiatives/csi/defining-social-innovation

2 defined as a solution (process, product, practice, market mechanism) implemented through diverse organizational models, developed by a range of actors in response to a systemic health challenge within a geographic context, enabling healthcare to be more inclusive, effective and affordable

3 https://socialinnovationinhealth.org/

## References

[1] Marmot M, Friel S, Bell R, Houweling TA, Taylor S (2008). Closing the gap in a generation: health equity through action on the social determinants of health. Lancet.

[2] Wilcox B, Aguirre A, Horwitz P. Ecohealth: Connecting Ecology, Health and Sustainability. New Dir Conserv Med Appl Cases Ecol Health. Aguirre AA., Ostfeld RS., Daszak P. Oxford: Oxford University Press; 2012.

[3] Wilcox BA, Aguirre AA, De Paula N, Siriaroonrat B, Echaubard P (2019). Operationalizing one health employing social-ecological systems theory: lessons from the greater Mekong sub-region. Front Public Health.

[4] Wilcox BA, Echaubard P, de Garine-Wichatitsky M, Ramirez B (2019). Vector-borne disease and climate change adaptation in African dryland social-ecological systems. Infect Dis Poverty.

[5] Duboz R, Echaubard P, Promburom P, Kilvington M, Ross H, Allen W, et al. Systems thinking in practice: participatory modeling as a Foundation for Integrated Approaches to health. Front Vet Sci. 2018;5:303. 10.3389/fvets.2018.00303 PMC6305083 30619895

[6] Farmer J, Carlisle K, Dickson-Swift V, Teasdale S, Kenny A, Taylor J (2018). Applying social innovation theory to examine how community co-designed health services develop: using a case study approach and mixed methods. BMC Health Serv Res.

[7] Mason C, Barraket J, Friel S, O’Rourke K, Stenta C-P (2015). Social innovation for the promotion of health equity. Health Promot Int.

[8] Gubler DJ (2011). Dengue, urbanization and globalization: the unholy trinity of the 21st century. Trop Med Health.

[9] World Health Organization. Dengue guidelines for diagnosis, treatment, prevention and control : new edition: World Health Organization; 2009. https://apps.who.int/iris/handle/10665/44188. Accessed 20 Dec 2019. 23762963

[10] Bhatt S, Gething PW, Brady OJ, Messina JP, Farlow AW, Moyes CL (2013). The global distribution and burden of dengue. Nature..

[11] World Health Organization (2019). Dengue Situation Updates 2019.

[12] Hahn H, Chastel C (1970). Dengue in Cambodia in 1963. Am J Trop Med Hyg.

[13] Huy R, Buchy P, Conan A, Ngan C, Ong S, Ali R (2010). National dengue surveillance in Cambodia 1980-2008: epidemiological and virological trends and the impact of vector control. Bull World Health Organ.

[14] Beauté J, Vong S (2010). Cost and disease burden of dengue in Cambodia. BMC Public Health.

[15] Huy R, Wichmann O, Beatty M, Ngan C, Duong S, Margolis HS (2009). Cost of dengue and other febrile illnesses to households in rural Cambodia: a prospective community-based case-control study. BMC Public Health.

[16] Vong S, Goyet S, Ly S, Ngan C, Huy R, Duong V (2011). Under-recognition and reporting of dengue in Cambodia: a capture-recapture analysis of the National Dengue Surveillance System. Epidemiol Infect.

[17] Wichmann O, Yoon I-K, Vong S, Limkittikul K, Gibbons RV, Mammen MP (2011). Dengue in Thailand and Cambodia: an assessment of the degree of underrecognized disease burden based on reported cases. PLoS Negl Trop Dis.

[18] Boyer S, Lopes S, Prasetyo D, Hustedt J, Sarady A, Dyna D (2018). Resistance of *Aedes aegypti* (Diptera: Culicidae) populations to deltamethrin, permethrin, and temephos in Cambodia. Asia Pac J Public Health.

[19] Khun S, Manderson L (2007). Community and school-based health education for dengue control in rural Cambodia: a process evaluation. PLoS Negl Trop Dis.

[20] World Health Organization (2013). Regional Office for the Western Pacific. Managing regional public goods for health : community-based dengue vector control.

[21] Hustedt J. Efficacy of guppies, community engagement and pyriproxyfen on dengue vectors in Cambodia: a cluster randomized trial. Joint Int Trop MedMeeting. 2015;2015 https://www.malariaconsortium.org/resources/publications/671/. Accessed 20 Dec 2019.

[22] Hustedt J, Doum D, Keo V, Ly S, Sam B, Chan V (2017). Determining the efficacy of guppies and pyriproxyfen (Sumilarv® 2MR) combined with community engagement on dengue vectors in Cambodia: study protocol for a randomized controlled trial. Trials..

[23] Wangroongsarb Y (1997). Dengue control through schoolchildren in Thailand.

[24] Barrera R, Amador M, Acevedo V, Caban B, Felix G, Mackay AJ (2014). Use of the CDC autocidal gravid ovitrap to control and prevent outbreaks of *Aedes aegypti* (Diptera: Culicidae). J Med Entomol.

[25] Barrera R, Amador M, Acevedo V, Hemme RR, Félix G (2014). Sustained, area-wide control of *Aedes aegypti* using CDC autocidal gravid ovitraps. Am J Trop Med Hyg.

[26] Barrera R, Acevedo V, Felix GE, Hemme RR, Vazquez J, Munoz JL (2017). Impact of autocidal gravid ovitraps on Chikungunya virus incidence in *Aedes aegypti* (Diptera: Culicidae) in areas with and without traps. J Med Entomol.

[27] Overgaard HJ, Alexander N, Matiz MI, Jaramillo JF, Olano VA, Vargas S (2016). A cluster-randomized controlled trial to reduce diarrheal disease and dengue entomological risk factors in rural primary schools in Colombia. PLoS Negl Trop Dis.

[28] Shafique M, Lopes S, Doum D, Keo V, Sokha L, Sam B (2019). Implementation of guppy fish (*Poecilia reticulata*), and a novel larvicide (pyriproxyfen) product (Sumilarv 2MR) for dengue control in Cambodia: a qualitative study of acceptability, sustainability and community engagement. PLoS Negl Trop Dis.

[29] World Health Organization (2012). Handbook for integrated vector management.

[30] Wallerstein N, Bernstein E (1988). Empowerment education: Freire’s ideas adapted to health education. Health Educ Q.

[31] Alamo-Hernandez U, Espinosa-García AC, Rangel-Flores H, Farías P, Hernández-Bonilla D, Cortez-Lugo M (2019). Environmental health promotion of a contaminated site in Mexico. EcoHealth.

[32] Sulistyawati S, Dwi Astuti F, Rahmah Umniyati S, Tunggul Satoto TB, Lazuardi L, Nilsson M (2019). Dengue vector control through community empowerment: lessons learned from a community-based study in Yogyakarta, Indonesia. Int J Environ Res Public Health.

[33] Nichter M, Nichter M, Thompson PJ, Shiffman S, Moscicki A-B (2002). Using qualitative research to inform survey development on nicotine dependence among adolescents. Drug Alcohol Depend.

[34] Gittelsohn J, Steckler A, Johnson CC, Pratt C, Grieser M, Pickrel J (2006). Formative research in school and community-based health programs and studies: “state of the art” and the TAAG approach. Health Educ Behav Off Publ Soc Public Health Educ.

[35] Mackay AJ, Amador M, Barrera R (2013). An improved autocidal gravid ovitrap for the control and surveillance of *Aedes aegypti*. Parasit Vectors.

[36] Mulgan G (2006). The process of social innovation. Innov Technol Gov Glob.

[37] Pol E, Ville S (2009). Social innovation: buzz word or enduring term?. J Socio-Econ.

[38] Walley J, Lawn JE, Tinker A, de Francisco A, Chopra M, Rudan I (2008). Primary health care: making Alma-Ata a reality. Lancet Lond Engl.

[39] Freire P (2000). Pedagogy of the oppressed. 30th anniversary ed.

[40] Westley F, Olsson P, Folke C, Homer-Dixon T, Vredenburg H, Loorbach D (2011). Tipping toward sustainability: emerging pathways of transformation. AMBIO..

