# Supplementary figures and images for: Fostering social innovation and building adaptive capacity for dengue control in Cambodia: a case study
Source: Infect Dis Poverty. 2020 Sep 3;9:126. doi: 10.1186/s40249-020-00734-y (PMC7469325; doi:10.1186/s40249-020-00734-y)

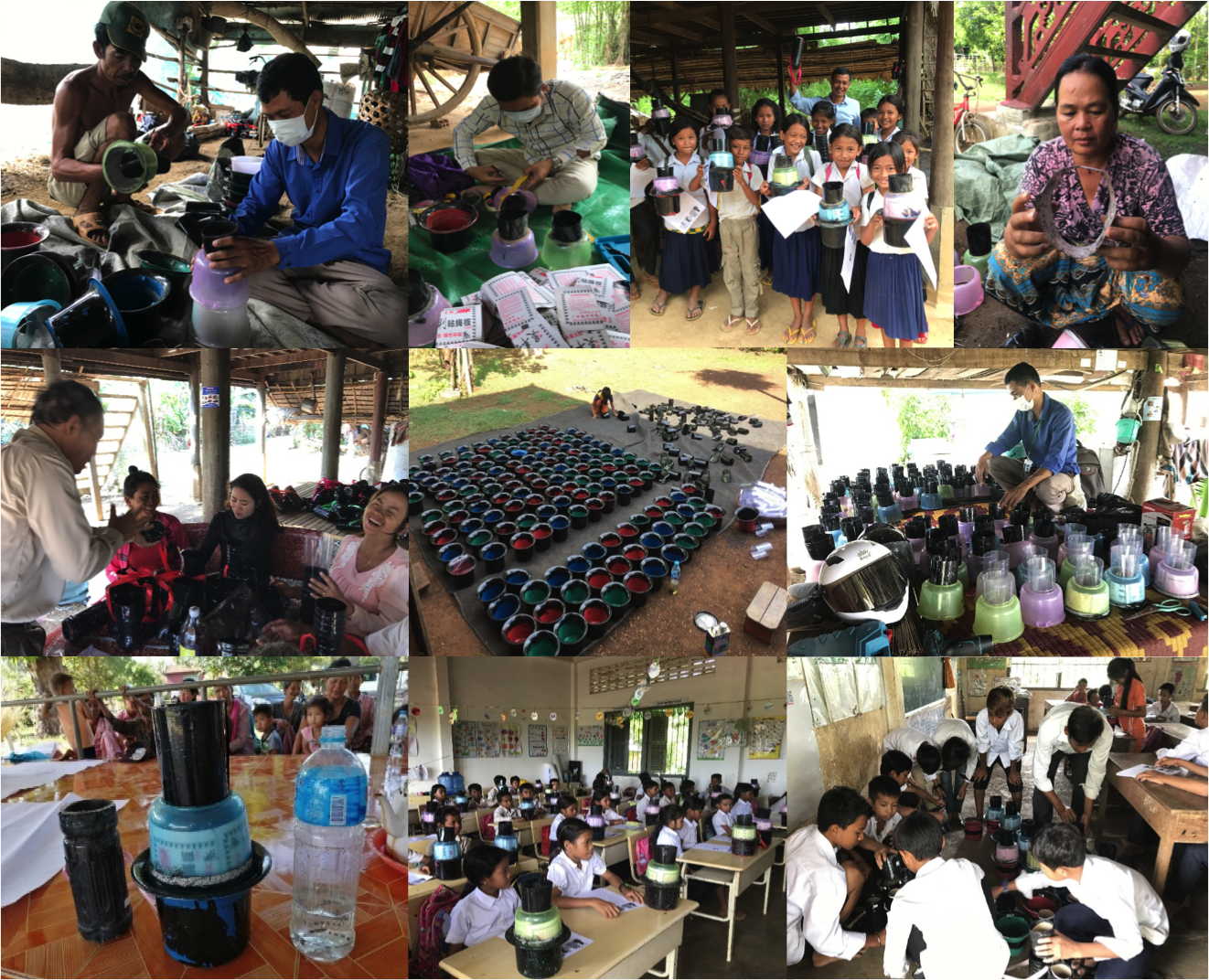

Supplement: Supplementary file 1 — Additional file 1. Trap production system and process of community inclusion. The upper left corner image shows an interaction with a local craftsman helping with traps design adaptation; continuing right and down to the lower rightmost corner, the images show women group’s involvement in production of large enough numbers of traps to be used in intervention schools and communities as well as school children involvement in trap deployment and use. [file 40249_2020_734_MOESM1_ESM.png]

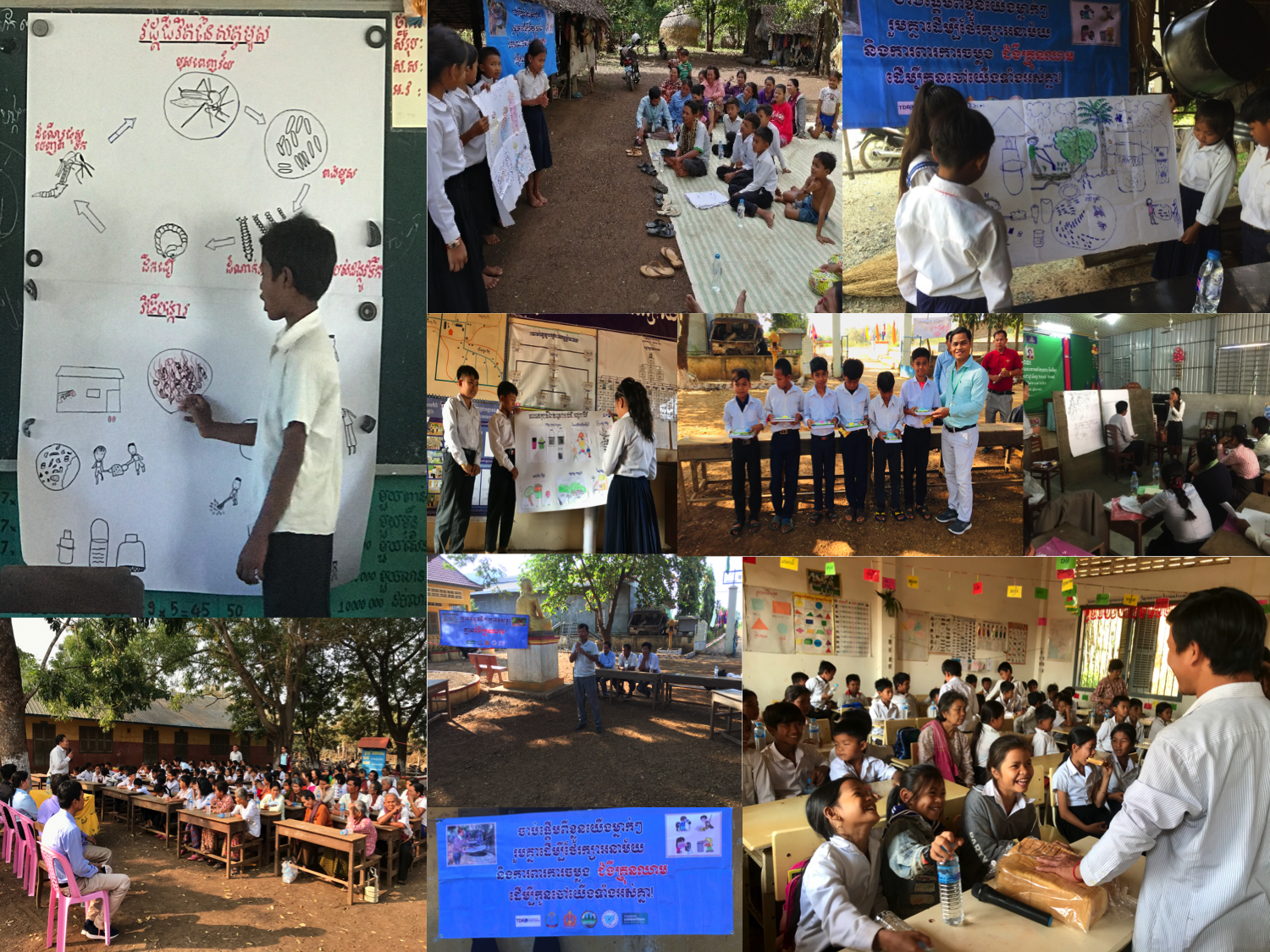

Supplement: Supplementary file 2 — Additional file 2. School-based and student led communication of Dengue information. [file 40249_2020_734_MOESM2_ESM.png]

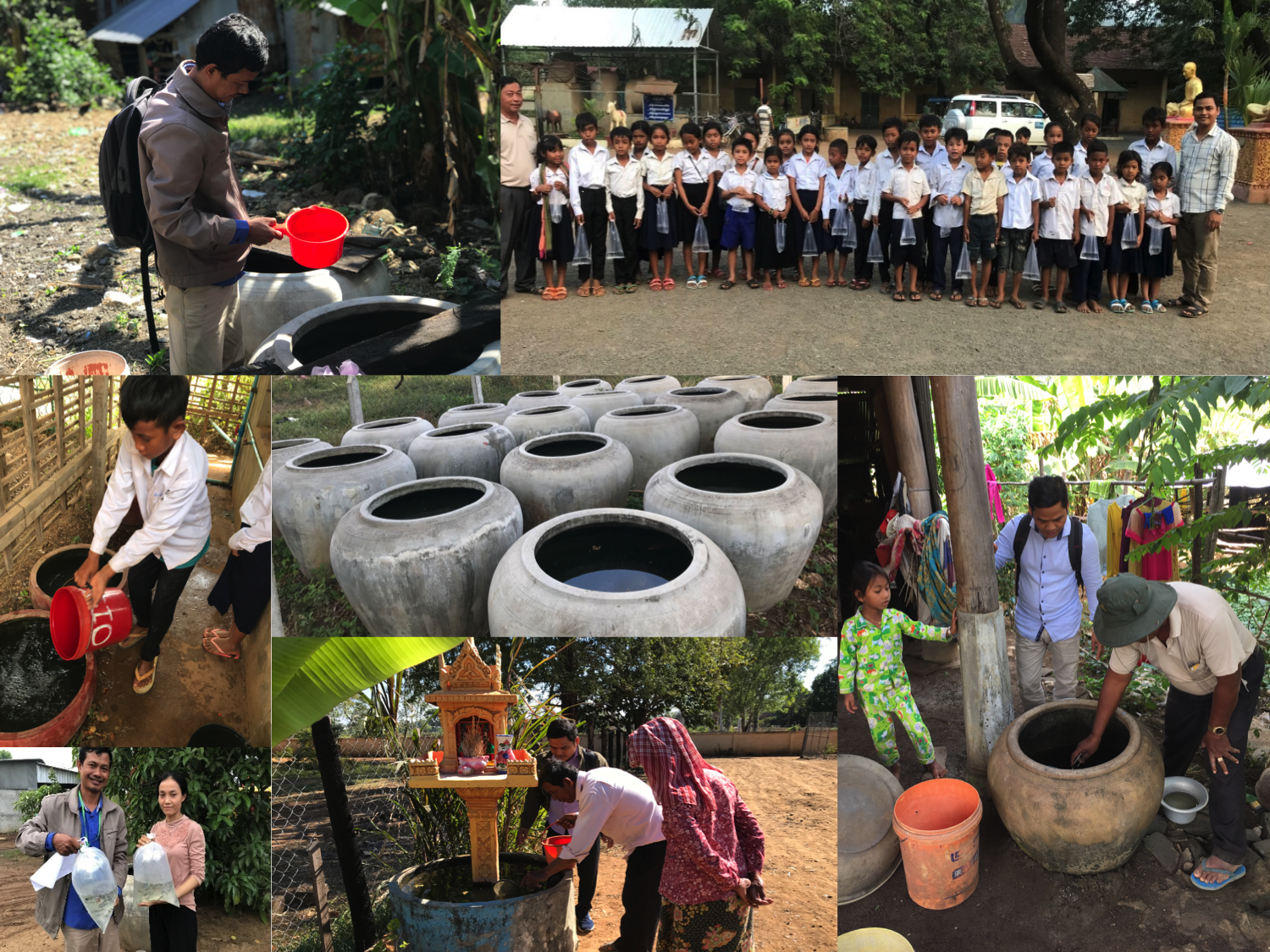

Supplement: Supplementary file 3 — Additional file 3. Guppy fish distribution network. [file 40249_2020_734_MOESM3_ESM.png]

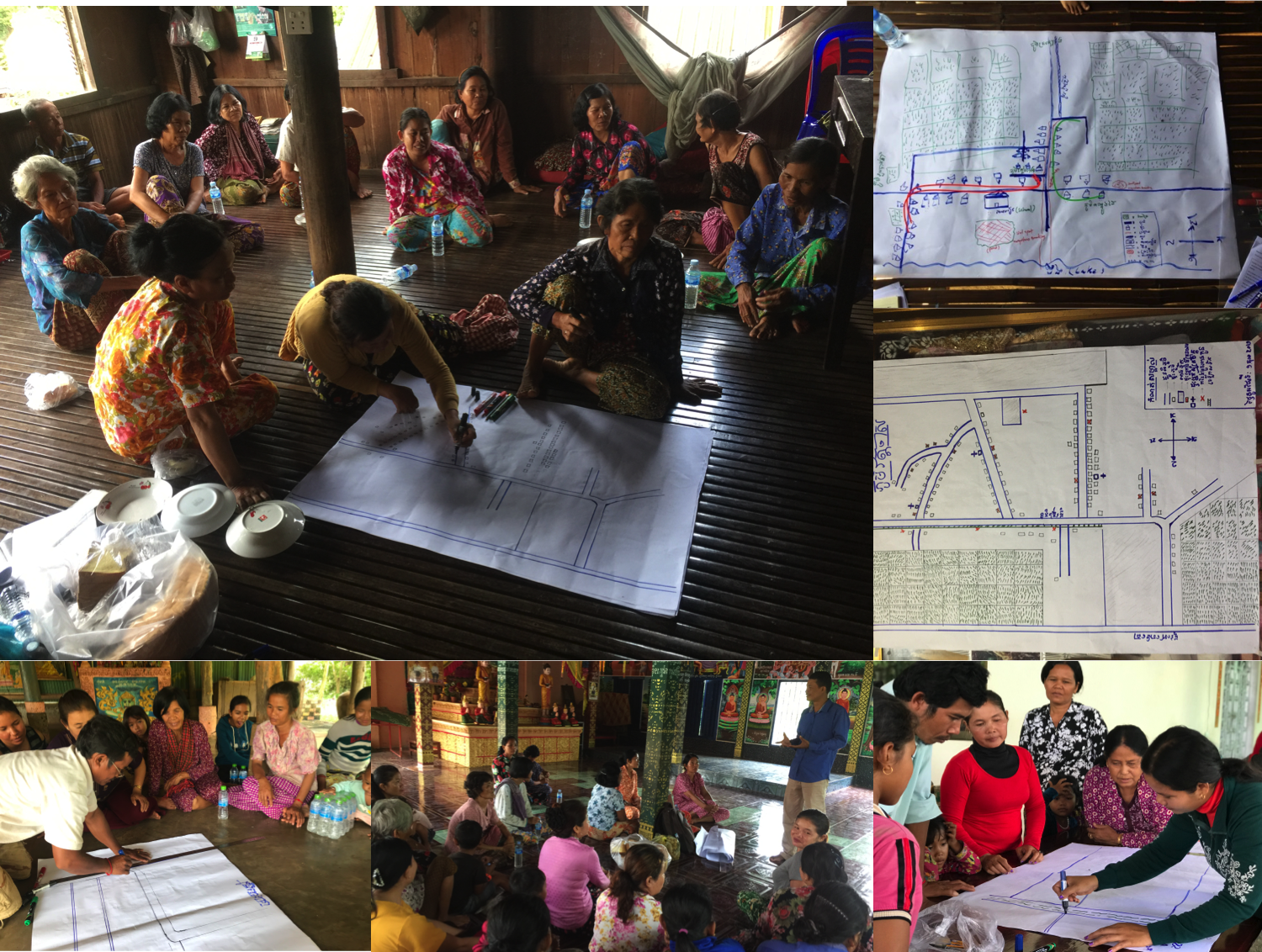

Supplement: Supplementary file 4 — Additional file 4. Participatory Epidemiology Mapping. [file 40249_2020_734_MOESM4_ESM.png]
